# The implications of an incidental chronic lymphocytic leukaemia in a resection specimen for colorectal adenocarcinoma

**DOI:** 10.1186/1477-7819-5-126

**Published:** 2007-10-30

**Authors:** Robert J Dennis, Justin C Alberts

**Affiliations:** 1 Department General Surgery, West Suffolk Hospital, Hardwick Lane, Bury St Edmunds, Suffolk IP33 2QZ. UK

## Abstract

**Background:**

Colorectal cancer and B cell chronic lymphocytic leukaemia (CLL) have a significant incidence, which are increasing with the aging population. Evidence has been presented in the literature to suggest that the synchronous presentation of colorectal cancer and B cell CLL may be more than simply coincidental for these two common malignancies. We report an unusual case of a presumed B cell CLL diagnosed on the basis of histological analysis of lymph nodes recovered from a resection specimen for rectal adenocarcinoma. We considered aetiological factors which may have linked the synchronous diagnosis of the two malignancies and the potential implications for the natural history of the two malignancies on one another.

**Case presentation:**

A 70-year-old male underwent low anterior resection with total mesorectal excision for a rectal adenocarcinoma. His co-morbid conditions were chronic obstructive airways disease and ischaemic heart disease. General examination revealed no lymphadenopathy. Full blood count, urea and electrolytes and liver function tests were all within normal limits. As well as confirming a pT3 N1 adenocarcinoma, histological analysis showed lymph nodes with an infiltrate of small lymphoid cells. Immunohistochemical studies showed these cells to be in keeping with B cell CLL.

**Conclusion:**

Whilst unable to identify any common aetiological factors in the two malignancies in our patient, immunosuppression and genetic abnormalities have been identified as possible bases for an observed epidemiological association between colorectal cancer and haematological malignancies. Examples such as our case of synchronous diagnosis of two malignancies in a patient are likely to increase with the aging population. The potential affects of one malignancy on the natural history of the other warrants further study. In our case, we considered that slow progression of the B cell CLL may increase the risk of recurrent rectal adenocarcinoma.

## Background

Colorectal cancer is the third commonest malignancy in Europe and North America. B cell chronic lymphocytic leukaemia (CLL) accounts for 0.8% of all cancers at any point in time [1]. One of the complications of B cell CLL is an associated increased risk of subsequent second malignancies, most commonly melanoma, soft-tissue sarcoma and colorectal and lung carcinoma [2-4]. Immunosuppression associated with the long term nature of B cell CLL has been proposed as the basis of this increased risk [5,6].

Whilst patients with advanced B cell CLL may experience weight loss, recurrent infections, bleeding secondary to thrombocytopenia, and/or anaemia, 25% of patients are asymptomatic and diagnosed on the basis of peripheral blood markers. Asymptomatic B cell CLL, diagnosed on peripheral blood markers or symptomatic presentation of B cell CLL has been described synchronously with rectal cancer [7]. We report an unusual case of a presumed B cell CLL diagnosed on the basis of histological analysis of lymph nodes recovered from a resection specimen for rectal adenocarcinoma. We considered the diagnostic quandaries of coincidental findings at examination of colorectal resection specimens; the possibility that a synchronous diagnosis may reflect an aetiological link between the malignancies in our patient; the possibility that the natural history of either one malignancy could be altered by the presence or treatment of the other.

## Case presentation

A 70-year-old male presented with an eight month history of looser stools. His medical history included a right lung lobectomy for tuberculosis aged 20, chronic obstructive airways disease and ischaemic heart disease. He was a long-term smoker.

General examination showed him to be well with no lymphadenopathy. No mass was palpable on digital rectal examination. Full blood count, urea and electrolytes and liver function tests were all within normal limits.

Flexible sigmoidoscopy and biopsy identified a rectal adenocarcinoma at 8 cm from the anal margin. MRI showed a normal liver and no para-aortic lymphadenopathy. A moderately bulky tumour was identifiable in the mid/low rectum, 5 cm in length. Local staging with MRI was T2 although the tumour was noted to be very close to the levator plate especially on the left. Perirectal and presacral lymph nodes were seen, the largest of which was 1 cm in short axis. CT scan of the chest did not identify any metastatic disease.

At laparotomy no peritoneal disease, liver metastases or abnormal lymph nodes were seen. A low anterior resection with total mesorectal excision was performed. Recovery was complicated by chest sepsis and prolonged ileus and the patient was fit for discharge on day 14 post surgery.

### Pathologic findings

Microscopic examination showed complete resection margins of a moderately differentiated adenocarcinoma extending through the muscularis propria. Metastatic adenocarcinoma was also identified in one of the twenty one lymph nodes. By UICC staging this was therefore pT3 N1 MX. There was no evidence of extramural vascular invasion. Several lymph nodes showed partial effacement of the normal lymphoid architecture by an infiltrate of small lymphoid cells in which many pseudoproliferation centres were evident.

### Immunohistochemical staining

Immunohistochemical studies confirmed the presence of CD20 positive B lymphoid infiltrate that co-expressed CD5, CD23 and BCL2 and was negative for cyclin D1, CD10 and BCL6 (DakoCytomation, Ely, UK).

This overall picture was considered consistent with a low grade B cell chronic lymphocytic leukaemia.

## Discussion

The meticulous examination of lymph nodes in colorectal resection specimens is crucial to evaluating prognosis and planning adjuvant therapies. On rare occasions such examinations will inevitably discover coincidental pathologies, presenting pathologists and clinicians with diagnostic, prognostic and therapeutic quandaries. This case was an unusual synchronous diagnosis of a presumed early B cell CLL and adenocarcinoma of the rectum. It presented the quandaries of the diagnosis of B cell CLL, the possibility of a link between the two malignancies and potential implications for the natural history of either malignancy.

In this case the histological and immunohistochemical findings were considered sufficient for a provisional diagnosis of B cell CLL. A formal diagnosis of B cell CLL could not be made since the patient did not have a lymphocytosis and a bone marrow biopsy was not considered appropriate at this stage of presentation. In modern medical practice these diagnostic quandaries need to be carefully discussed with the patient. However it is all too easy for confusion to develop amongst the various medical staff involved in a patient's care, the patient and their relatives.

The most likely explanation for this synchronous diagnosis of a presumed B cell CLL and adenocarcinoma of the rectum is purely coincidental. However in the synchronous presentation of colorectal cancer and B cell CLL an epidemiological association has been noted which goes further than simple coincidence [8]. This synchronous relationship has been explained in terms of immunosuppression over a prolonged period due asymptomatic B cell CLL and a subsequent development of colorectal cancer, in fact, in a metachronous manner. This is consistent with the observed increase in the long term risk of solid organ malignancies in a patient diagnosed with B cell CLL [2,3], where immunosuppression over a prolonged period has also been postulated as the basis for the increased risk [5,6]. Given the very early stage of the presumed B cell CLL in our patient it would seem unlikely that immunosuppression has played a significant role in the development of the primary rectal carcinoma.

An alternative explanation for the link between colorectal cancer and B cell lymphocytic leukaemia may come from our increasing understanding of tumour genetics. A common alteration in a locus of chromosome 7 has been suggested as a genetic basis for an association between haematological malignancy and hereditary nonpolyposis colon cancer [9]. Leukaemia and colorectal cancer are both associated with Li-Fraumeni syndrome and mutations of the p53 gene/gene expression. Abnormalities in the p53 gene expression have been demonstrated in 10–15% of B cell CLL patients and 75% of colorectal carcinoma patients. In B cell CLL p53 gene expression has been shown to be a marker of aggressive disease with a poorer prognosis [10]. With the exception of Li-Fraumeni syndrome, single gene mutations would not account for the development of both malignancies in a single patient. However altered p53 expression may illustrate common pathways, many of which remain unknown, which may increase an individual's risk of more than one form of malignancy. As a matter of interest we performed immunohistochemistry for p53 protein expression in our patient's histopathological specimens and identified p53 expression in the cells of the adenocarcinoma, but not the B cells of the CLL.

In the synchronous diagnosis of B cell CLL and rectal carcinoma, as well as implying the possibility of common aetiological factors, we also considered whether either disease or its treatment might affect the natural history of the other. This patient had pT3 N1 stage rectal adenocarcinoma and a predicted 5 year survival in of 68% – 62% [11,12]. On the basis of the N1 stage disease the patient was offered adjuvant chemotherapy. As first line chemotherapy the patient received a course of 5-fluorouracil. There is no evidence to suggest that administration of 5-fluorouracil alters the natural history of B cell CLL.

Variability in the rate of B cell CLL disease progression and incidence of disease related complications can make the decision to treat a more complex one. Treatment of early stage B cell CLL with chemotherapy does not confer any survival advantage over conservative management. Treatment of B cell CLL also does not reduce the incidence of second neoplasia [13]. Given the very early stage of a presumed B cell CLL in our patient we decided to monitor him until evidence of disease progression. However with slow progression of the B cell CLL the patient may become relatively immunosuppressed for a prolonged period before treatment is indicated. Given the established increased risk of primary colorectal cancer potentially associated with this situation it is of concern that our patient may be at increased risk of recurrent rectal carcinoma in the future. The risk of recurrent solid organ tumours with a diagnosis of B cell CLL has not been studied. In our case any increased risk of recurrent rectal carcinoma would potentially alter the duration of surveillance. Surveillance in our hospital for rectal carcinoma is 5 years with annual CT chest and abdomen. With a predicted median survival of >7 years with the B cell CLL surveillance beyond 5 years may be prudent.

## Conclusion

The meticulous examination of lymph nodes in colorectal carcinoma can reveal other unsuspected malignancies. We have described an unusual case of synchronous rectal adenocarcinoma and a presumed early B cell CLL diagnosed on the basis of histological examination of the rectal resection specimen. Whilst unable to identify any common aetiological factors in the two malignancies in our patient, immunosuppression and common tumour genetics may form the bases for an observed epidemiological association between colorectal cancer and haematological malignancies. Examples such as our case of synchronous diagnosis of two malignancies in a patient are likely to increase with the aging population. The potential affects of one malignancy on the natural history of the other warrants further study. In our case, we considered that slow progression of the presumed B cell CLL may increase the risk of recurrent rectal adenocarcinoma and prolonged surveillance may be prudent.

## Competing interests

The author(s) declare that they have no competing interests.

## Authors' contributions

**JA **was the operating surgeon and consultant overseeing the patient's care. **RD **and **JA **were responsible for drafting the manuscript and revising it critically. All authors read and approved the final manuscript.
